# Expert Consensus of Syndrome Differentiation for Phlegm Turbidity Syndrome for Coronary Heart Disease

**DOI:** 10.1155/2018/8184673

**Published:** 2018-12-02

**Authors:** Xin-lin Chen, Xiao-qi Liu, Rong Xie, Dan-hong Peng, Yan-ping Wang, Xuan Zhou, Bin Wang, Chuan-wei Mo, Qian Xu, Xiantao Li

**Affiliations:** School of Basic Medical Science, Guangzhou University of Chinese Medicine, Guangzhou, China

## Abstract

**Objective:**

The purpose of the study was to form a questionnaire of expert consensus about phlegm turbidity syndrome of coronary heart disease (CHD) using literature method and Delphi method, which could provide the objective evidences for the clinical diagnosis and treatment for CHD.

**Method:**

The CBM, CNKI, VIP, and PubMed were searched. The articles about phlegm turbidity syndrome for CHD with the definite related four diagnostic data were included. Based on the results of the literature method, two rounds of Delphi method were conducted. The TCM experts about CHD were enrolled. Concentration and coordination index of the experts were used to select the items.

**Results:**

Literature method: A total of 118 articles were included. Greasy fur, slippery pulse, chest fullness or chest pain, anorexia, nausea and vomiting, vertigo, excessive phlegm, abdominal fullness, head heaviness, obesity, stringy pulse, physical heaviness, soft pulse, somnolence, fatigue, and pale tongue (16 items) had the relatively high proportion, and they were eligible for Delphi process. Delphi method: A total of 93 experts (22 for the first round, 71 for the second round) were included. The reliability of the items was 0.885 for all the experts. The 16 items were not significantly different between the two rounds (P>0.05). According to the results of mean, frequency, sum of ranks and coefficient of variation, the item of nausea and vomiting, somnolence, pale tongue, and soft pulse were deleted.

**Conclusions:**

The questionnaire of phlegm turbidity syndrome of CHD was established, with good reliability. The sensitivity and specificity of the questionnaire are still necessary to further validate for clinical or scientific use.

## 1. Introduction

Coronary heart disease (CHD: coronary artery disease) was one of the most common chronic diseases all over the world. The epidemic of CHD was effectively confined to North America, the relatively rich countries of Europe, Australia, and New Zealand [1–4]. Since the 21st century, CHD was epidemic in the newly industrializing and other “non-western” countries, such as Eastern European countries, Latin America, Southeast Asia, India, and sub-Saharan Africa [3–5]. CHD is the 4th leading cause of mortality in China [6]. China is facing the current situation of rapid aging of the population. Some experts predict that there will be 7.8 million excess CHD (a 69% increase) and 3.4 million excess CHD deaths (a 64% increase) in 2020–2029 compared with 2000–2009 in Chinese [7].

Previous studies documented that traditional Chinese medicine (TCM) had potential advantages in the treatment of the CHD patients [8–11]. Some researchers suggested that for CHD, Qi and Yin deficiency syndrome gradually reduced, and phlegm turbidity syndrome (PTS) increased in the past 40 years [12, 13]. The PTS had become one of main TCM syndromes for the CHD patients [14]. According to the theories of TCM, the four diagnostic methods (syndrome differentiation) of TCM could provide an effective basis for the diagnosis and treatment of the PTS [15, 16].

The PTS standards of CHD were studied widely [17]. Five standards were established by the researchers [18–22]. However, there was a lack of the uniform PTS standard for CHD. The five standards had some differences items. The PTS is directly diagnosed by the TCM practitioners using their nude eyes in inspection, or fuzzy qualitative words in interrogation. As a result, the diagnostic results are inclined to be inconsistent among different TCM experts. What is more, as the life style changes, the clinical manifestations of the PTS also change. Thus the standard of phlegm turbidity syndrome should be changed accordingly. Therefore, it is important and urgent to establish the uniform PTS diagnosis standard for CHD.

The standard procedures for developing quality of life (QOL) questionnaires were followed to develop and validate the PTS questionnaire for CHD [23–27]. The procedures included construct definition, item generation, pilot study, and validation study. We aim to develop the PTS questionnaire for the CHD patients and hope that the results can provide the evidences for the clinical diagnosis and treatment. In this study, we focus on the construct definition and item generation of the PTS questionnaire.

## 2. Methods

The research team was composed of two CHD physicians, two TCM diagnostics educators and two QOL researchers. The team was responsible for the design, implementation, and statistical analysis of the study. The literature method and Delphi method were used to definite the construct of the PTS questionnaire and generate the item.

Spleen governs the transportation of water in the theories of TCM [28]. What is more; spleen has the function to turn food into nutritive refined substance, and transport these substances to the whole body. When the function of spleen is deficient or loss, water and food cannot be transported or transformed normally, which results in phlegm turbidity [28]. When phlegm turbidity exists, the QI is obstructed and the orifices are confused. Therefore, the CHD patients with PTS usually have the following symptoms: headache, chest pain, chest fullness, excessive phlegm, nausea, vomiting, greasy fur, fat tongue, and slippery pulse.

### 2.1. Literature Method

The literature method was used to establish the items pool of phlegm turbidity syndrome for CHD. A literature search was conducted from their inceptions to July 8th, 2015, in the following databases: Chinese biomedical literature database (CBM), China National Knowledge Infrastructure (CNKI), VIP, and PubMed. The search terms included the following terms:

(1) “phlegm turbidity syndrome” OR “phlegm damp syndrome” OR “phlegm syndrome” OR “phlegm-turbid syndrome” OR “phlegm impotency syndrome” OR “phlegm stasis syndrome”

(2) “standard” OR “criterion” OR “diagnosis” OR “syndrome differentiation”

(3) “coronary heart disease” OR CHD OR “coronary artery disease” OR CAD OR coronary disease

(4) #1 AND #2 AND #3

The inclusion criteria comprised the following two aspects: (1) the articles were included if it contained the PTS standards for CHD and (2) the articles had the definite related four diagnostic data (including symptoms, tongue and pulse). The articles or the reviews without the PTS standards, the article or the reviews about etiology and pathogenesis of the PTS, and the article about animal experiment were excluded. The articles published in abstract were considered only if sufficient data could be retrieved from the abstract or following contact with the authors.

Duplicate articles from different databases were identified, and the remaining abstracts were read for eligibility. The full texts of the articles were retrieved and reviewed independently by two authors. Any disagreements were resolved by the consensus of group discussion.

The terminology of PTS and its four diagnostic data for CHD were standardized according to Chinese medicine clinical diagnosis and treatment terminology of People's Republic of China, common terms in Chinese Medicine, Dictionary of traditional Chinese Medicine, and National standard terminology of People's Republic of China. For example, poor appetite, loss of appetite, inappetence, eating less, and decrease of diet were all classified as “anorexia.” The four diagnostic data (item) was collected from the included articles. The frequency of four diagnostic data was calculated. At last, the items pool was formed, and the most common items are chosen which was used in Delphi method.

### 2.2. Delphi Method

The Delphi method was used to select the items for the PTS questionnaire and determine its corresponding weight. The Research Ethics Committee of Guangzhou University of Chinese Medicine provided ethical approval for the Delphi study. Eligibility criteria of the experts included (1) the doctors who had rich clinical experience of CHD (>3 years), with the major of TCM or Integrative Chinese and western medicine (ICWM); and (2) who provide informed consent to participate. The experiences, geography, and profession of the experts were considered to ensure more comprehensive opinions of different experts. The experts were excluded if the doctors did not work on CHD or their major was Western Medicine.

Before Delphi study, the investigators received uniform training to be familiar with the questionnaire. If the experts did not understand the meaning of the items from the questionnaire, the investigators should give appropriate explanations. All the experts independently answered the items of the questionnaire. They were allowed to modify the item which may be not understood easily and add the new items.

**Figure 1:**
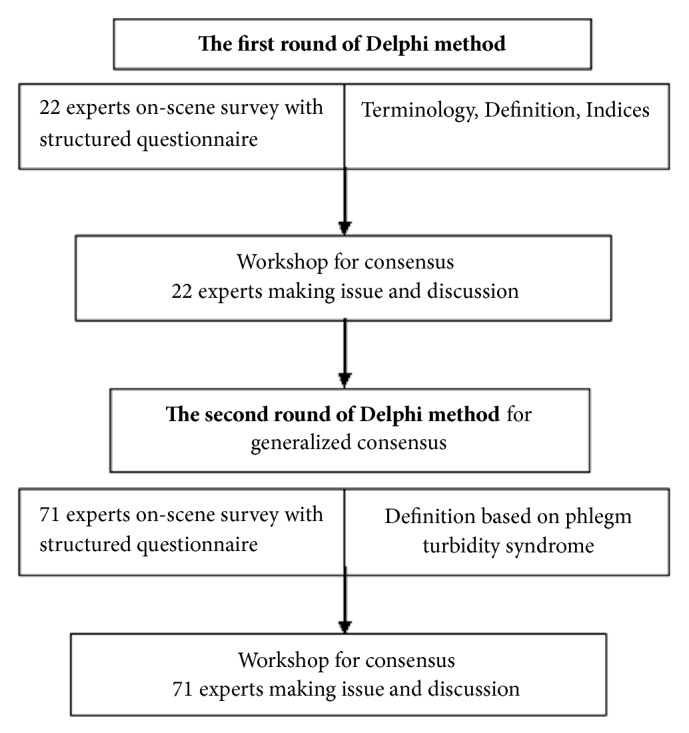
Summary of two-round Delphi method in this study.

The study was conducted between 1 July 2015 and 31 October 2015. Two rounds of Delphi method were launched for formulating a main differentiation and engaged in based consensus in the first round and producing a generalized agreement in the second round (Figure 1). All the experts in the first round and the second round were enrolled in the national heart conference. The first round questionnaire was answered by 22 experts to confirm indication. The results of the first round were summarized and each expert was given the summary ranked from 1 (not at all) to 5 (very important). Discussion issues on this subject were defined via workshop based on the results of the first round Delphi method. The items of terminology and definition were defined by the experts. In the second round, questionnaire was answered by 71 experts to support reliability and validity. The experts were revised their ranking based on summarized from round 2.

The degree of concentration of the experts included mean of the item, the frequency of full score (very important), and sum of ranks. The greater mean of the item, the more frequency of full score and the lower scores of sum of ranks meant that the item was more important. It also indicated the more concentrated to the item for the experts. The coordination degree is measured by coefficient of variation, which reflected the degree of variation of the experts. If coefficient of variation is less than 0.25, it meant that the item is good. The overall coordination coefficient was also calculated.

Mean, frequency, sum of ranks, and coefficient of variation were used to select the important items. If the item had the indexes which was less than the standard, then the item was deleted [29]. If not, the team discussed the item together according to the theory of TCM in order to make a choice. SPSS 21.0 (Chicago, IL) was used for the analysis.

## 3. Results

### 3.1. Literature Method

**Figure 2:**
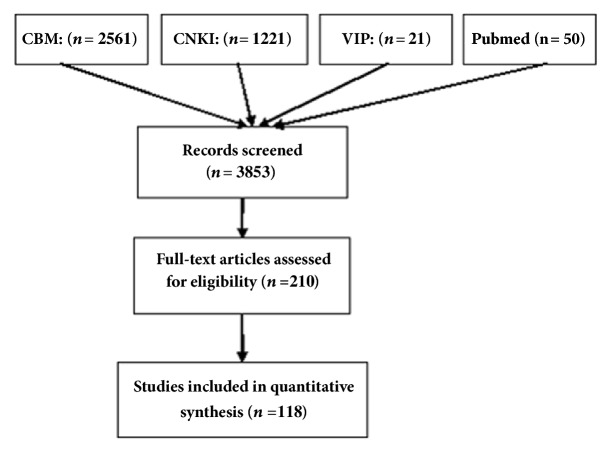
Flowchart of the search strategy.

A total of 3853 articles met the inclusion criteria (Figure 2). The titles and abstracts of all articles were reviewed. The full texts of the 210 studies were retrieved for review, and 118 studies were included for analysis.

There were a total of 83 symptoms for PTS. The results showed that greasy fur had the highest frequency (94.9%), followed by slippery pulse (89.0%). Greasy fur, slippery pulse, chest fullness or chest pain, anorexia, nausea and vomiting, vertigo, excessive phlegm, abdominal fullness, head heaviness, obesity, stringy pulse, physical heaviness, soft pulse, somnolence, fatigue, and pale tongue had the relative high proportion (Table 1). Based on the results of systematic literature reviews and several group discussion, 16 items was retained.

Five criteria of PTS for CHD were also searched (Table 2). (1) In 1980, the National Symposium of CHD established the standard of PTS. The clinical manifestations of PTS included chest fullness, nausea, palpitations, white slippery or greasy fur (partial cold), slippery pulse or irregularly intermittent pulse (partial cold), yellow greasy fur (hot), and stringy and slippery pulse or stringy and rapid pulse (hot)[18]. (2) In 1985, Zhang edited the Internal Medicine of TCM. The clinical manifestations of PTS contained chest tightness, chest pain, shoulder back pain, shortness of breath, physical heaviness, obesity, excessive phlegm, greasy fur, and slippery pulse [19]. (3) In 1994, State Administration of Traditional Chinese Medicine of the People's Republic of China released the Standard of Chinese medicine industry [20]. The clinical manifestations of PTS contained heart stuffy, shortness of breath, obesity, physical heaviness, abdominal fullness, excessive phlegm, greasy fur, and slippery pulse. (4) In 2002, the guiding principles of clinical research on new drugs of traditional Chinese Medicine showed that the clinical manifestations included oppression in chest, shoulder back pain, shortness of breath, obesity, excessive phlegm, physical heaviness, turbid and greasy fur or slippery fur, and slippery pulse [21]. (5) In 2008 [22], China association of traditional Chinese medicine and pharmacy reported that the clinical manifestations of PTS contained chest tightness, light heartache, physical heaviness, abdominal fullness, anorexia, nausea, sticky mouth, spit saliva, white and greasy fur or white and slippery fur, and slippery pulse.

### 3.2. Delphi Method

A total of 22 experts were enrolled in the first round of Delphi method and 71 experts in the second round. The characteristic of the experts was not significantly different between the two rounds (*P* > 0.05). The average ages of all the experts were 47.8 ± 10.0 years old, which range from 29 to 84. 52.7% of the experts were male (Table 3). Of the experts, 49 were professors and 22 were associate professor. All the experts were from coronary heart disease. 61.3% (57) were TCM, and 38.7% were ICWM.

All the items were not significantly different between the two rounds of Delphi method (P > 0.05, Table 4).

Basing on the combined results of Delphi method, most of the items were scored “very important” and “important” (Table 5). The items of greasy fur and chest fullness/chest pain had the highest proportion of “very important.” The item of pale tongue had the highest proportion of “less important.” The items of vertigo, somnolence, pale tongue, soft pulse, and stringy pulse had a low proportion of “not at all.” No items had the missing data.

### 3.3. Concentration and Coordination Index of the Experts

The overall coordination coefficient is 0.264, and Chi-square value is 649.1, with P < 0.001, which indicates that the expert consensus was good and the coordination degree was high. Cronbach's *α* of the first and second rounds were 0.905 and 0.876, respectively. Cronbach's *α* was 0.885 for all the experts. The contribution rate of cumulative variance was 63.21% for all the experts.

The concentration and coordination index of the experts were shown in Table 6. The mean scores of the items ranged between 3.39 and 4.76. The item of greasy fur had the highest score (4.76), followed by slippery pulse (4.54) and chest fullness/chest pain (4.43). The item of pale tongue had the lowest score (3.39), followed by soft pulse (3.48) and stringy pulse (3.59). The item of pale tongue had the highest score (243) for the sum of rank, followed by soft pulse (234) and stringy pulse (224). The item of greasy fur had the lowest score (127) for the sum of rank. The item of pale tongue had the highest score (0.298) for coefficient of variation, followed by stringy pulse (0.270) and soft pulse (0.267). The item had the lowest score (0.095) for coefficient of variation.

According to the suggestions of the experts, 4 other items were recommended: sticky skin, palpitation, sticky stools, and eating habits. The item of sticky stool was recommended by two experts. Other items were recommended by only one expert. The addictive four items were all deleted after the discussion of the group, because the frequency was too low.

At last, the items of nausea and vomiting, somnolence, pale tongue, and soft pulse were deleted. The item were greasy fur, slippery pulse, chest fullness or chest pain, anorexia, vertigo, excessive phlegm, abdominal fullness, head heaviness, obesity, stringy pulse, physical heaviness, somnolence, fatigue, and plump tongue. These items and their definition were shown in Table 7.

## 4. Discussion

Based on the theories of TCM, this study followed the standard procedures of QOL development to develop the items of PTS questionnaires for CHD [23–27]. The structures of PTS were formed. And then, the items of PTS were selected using the literature method and Delphi method to develop the first version of the PTS questionnaire.

Our results showed that chest fullness/chest pain, excessive phlegm, abdominal fullness, obesity, anorexia, head heaviness, vertigo, physical heaviness, fatigue, greasy fur, slippery pulse, and stringy pulse were included for the diagnose of phlegm turbid syndrome for CHD through literature method and Delphi method. All the items were the common and important symptoms for PTS of CHD. Our questionnaire had some difference from the previous standards. (1) Abdominal fullness, head heaviness, and vertigo were the additive items basing on the literature method and Delphi method. These items were not the important symptom in the previous standards. (2) The item “dim complexion” was not included in our new PTS standard, which was different from the previous standards. (3) The item “obesity” was included, because people in modern times ate more meat and did less physical exercises which resulted in the excess nutrition. According to the theory of TCM, the excess nutrition could produce excessive phlegm.

The items of nausea and vomiting, somnolence, soft pulse, and pale tongue were deleted from PTS after Delphi method, which was consistent with the theory of TCM. (1) Nausea and vomiting was not the common symptom for the CHD patients. What was more is that it was not the specific item for PTS. Qi-yin deficiency syndrome, heart-yang deficiency syndrome, and yin-yang deficiency syndrome also had the symptom of nausea and vomiting [16]. (2) The researcher reported that the symptom of somnolence could appear in turbid phlegm blocking syndrome, spleen-qi deficiency syndrome, yang-qi deficiency syndrome, and heart-qi deficiency syndrome. Somnolence was the most common in the phlegm dampness and blood stasis syndrome according to the investigation results [30]. Somnolence was not the main symptom for PTS. (3) The item of soft pulse mainly appears in deficiency syndrome or dampness syndrome, but not in PTS. (4) In modern times, people changed their eating habits. People ate more meat and did less physical exercises. Common diseases nowadays such as cardiovascular disease and diabetes were related to excess nutrition. According to the theory of TCM, the excess nutrition could produce excessive phlegm. Therefore, the symptom of “pale tongue” was not common for CHD patients with PTS. Therefore, “pale tongue” was not an important indicator for PTS.

Compared with the previous five standards [18–22], the items of the PTS questionnaire were described more clearly. For example, the item “excessive phlegm” in the previous five standards was not difficult to quantify. Excessive phlegm in our questionnaire was divided into three levels: (1) mild: 10-49 ml phlegm day and night, or 5-25 ml phlegm at midnight and dawn; (2) moderate: 50-100 ml phlegm day and night, or 26-50 ml phlegm at midnight and dawn; and (3) severe: more than 100 ml phlegm day and night, or more than 50 phlegm at midnight and dawn. In this study, two rounds of Delphi methods were conducted to select the items of the PTS questionnaire. The coordination coefficient of the two rounds of expert consultation was good, which indicated that the opinion among different experts tends to be consistent.

The literature method can provide an objective basis for Delphi process. It can improve the credibility of the results for expert consultation. These methods reduced the subjective factors, resulting in the reliable conclusions of the study. The literature research can help to select the items which could represent the characteristics of CHD patients with PTS in modern times.

The recovery rate of the questionnaire and the response rate of the experts were both 100% in Delphi method. It showed that our results of Delphi method were reliable. The results of literature method and Delphi method concluded that there was a certain degree of consistency, suggesting that the method of obtaining expert advice through the literature research method was desirable.

There were some limitations. (1) The results based on Delphi method were subjective, which was obviously affected by the included experts. However, the TCM or ICWM experts were all from Grade Three TCM Hospital and had rich clinical experience on the treatment of CHD. (2) The questionnaire of PTS should be further evaluated using the data of the CHD patients.

## 5. Conclusions

We established the PTS questionnaire of CHD, which can be used for clinical diagnosis and treatment. Our results showed that the PTS questionnaire had good reliability, with the clearly defined items. It was necessary to validate the questionnaire in clinical research and to confirm the sensitivity and specificity for clinical or scientific use.

## Figures and Tables

**Table 1:** The items of phlegm syndrome basing on literature search.

Item	Frequency (%)	Item	Frequency (%)
Greasy fur	112 (94.9)	Head heaviness	51 (43.2)
Slippery pulse	105 (89.0)	Obesity	42 (35.6)
Chest fullness chest pain	85 (72.0)	Stringy pulse	32 (27.1)
Anorexia	61 (51.7)	Physical heaviness	31 (26.3)
Nausea and vomiting	59 (50.0)	Soft pulse	28 (23.7)
Vertigo	58 (49.2)	Somnolence	27 (22.9)
Excessive phlegm	56 (47.5)	Fatigue	26 (22.0)
Abdominal fullness	52 (44.1)	Pale tongue	22 (18.6)

**Table 2:** Five previous standards of phlegm turbidity syndrome for CHD.

	1980 standard	1985 standard	1994 standard	2002 standard	2008 standard
Chest fullness	1	1	1	1	1
Chest pain	0	1	0	1	1
Nausea	1	0	0	0	1
Shortness of breath	0	1	1	1	0
Palpitations	1	0	0	0	0
Flustered	1	0	0	0	0
Physical heaviness	0	1	1	1	1
Abdominal fullness	1	0	1	0	1
Obesity	0	1	1	1	0
Excessive phlegm	0	1	1	1	1
Sticky mouth	0	0	1	0	1
Anorexia	0	0	0	0	1
Greasy/slippery fur	1	1	1	1	1
Slippery/stringy pulse	1	1	1	1	1

**Table 3:** The characteristic of the experts.

	The first round	The second round	*P* value	Total
Age ($\overset{-}{x}\pm s$)	49.6±12.2	47.5±9.7	0.407	47.8±10.0
Sex				
Male	12	37	0.842	49
Female	10	34		44
Professional title				
Professor	14	33	0.387	49
Associate professor	3	20		22
Lecturer	5	16		20
Assistant	0	2		2
Major				
TCM	13	44	0.808	57
ICWM	9	27		36

TCM: Traditional Chinese Medicine and ICWM: Integrative Chinese and western medicine.

**Table 4:** The distribution of the items in the first and second round (Delphi method).

Item	Round	Very important	Important	Median	Less important	Not at all	*P* value
Chest fullness/chest pain	The first	9	9	4	0	0	0.085
	The second	43	20	8	0	0	
Excessive phlegm	The first	10	5	7	0	0	0.906
	The second	29	26	15	1	0	
Abdominal fullness	The first	8	10	4	0	0	0.811
	The second	30	26	15	0	0	
Obesity	The first	8	10	4	0	0	0.700
	The second	21	39	8	3	0	
Anorexia	The first	3	13	4	2	0	0.902
	The second	18	26	24	3	0	
Nausea and vomiting	The first	4	6	11	1	0	0.649
	The second	12	28	25	6	0	
Head heaviness	The first	4	15	2	1	0	0.354
	The second	23	37	9	2	0	
Vertigo	The first	4	10	3	4	1	0.052
	The second	23	33	13	1	1	
Somnolence	The first	4	7	8	3	1	0.237
	The second	13	33	22	2	0	
Physical heaviness	The first	9	8	4	1	0	0.286
	The second	33	32	5	1	0	
Fatigue	The first	6	8	7	1	0	0.608
	The second	24	24	19	4	0	
Pale tongue	The first	2	7	7	5	1	0.312
	The second	13	18	29	10	1	
Greasy fur	The first	17	5	0	0	0	0.989
	The second	55	15	1	0	0	
Slippery pulse	The first	9	11	2	0	0	0.053
	The second	48	18	5	0	0	
Soft pulse	The first	4	6	8	3	1	0.682
	The second	8	29	26	7	1	
Stringy Pulse	The first	1	9	8	3	1	0.099
	The second	18	19	29	4	1	

**Table 5:** The frequency of the items (Delphi method).

	Very important (%)	Important (%)	Median (%)	Less important (%)	Not at all (%)
Chest fullness/chest pain	52 (55.9)	29 (31.2)	12 (12.9)	0 (0.0)	0 (0.0)
Excessive phlegm	39 (41.9)	31 (33.3)	22 (23.7)	1 (1.1)	0 (0.0)
Abdominal fullness	38 (40.9)	36 (38.7)	19 (20.4)	0 (0.0)	0 (0.0)
Obesity	29 (31.2)	49 (52.7)	12 (12.9)	3 (3.2)	0 (0.0)
Anorexia	21 (22.6)	39 (41.9)	28 (30.1)	5 (5.4)	0 (0.0)
Nausea and vomiting	16 (17.2)	34 (36.6)	36 (38.7)	7 (7.5)	0 (0.0)
Head heaviness	27 (29.0)	52 (55.9)	11 (11.8)	3 (3.2)	0 (0.0)
Vertigo	27 (29.0)	43 (46.2)	16 (17.2)	5 (5.4)	2 (2.2)
Somnolence	17 (18.3)	40 (43.0)	30 (32.3)	5 (5.4)	1 (1.1)
physical heaviness	42 (45.2)	40 (43.0)	9 (9.7)	2 (2.2)	0 (0.0)
Fatigue	30 (32.3)	32 (34.4)	26 (28.0)	5 (5.4)	0 (0.0)
Pale tongue	15 (16.1)	25 (26.9)	36 (38.7)	15 (16.1)	2 (2.2)
Greasy fur	72 (77.4)	20 (21.5)	1 (1.1)	0 (0.0)	0 (0.0)
Slippery pulse	57 (61.3)	29 (31.2)	7 (7.5)	0 (0.0)	0 (0.0)
Soft pulse	12 (12.9)	35 (37.6)	34 (36.6)	10 (10.8)	2 (2.2)
Stringy Pulse	19 (20.4)	28 (30.1)	37 (39.8)	7 (7.5)	2 (2.2)

**Table 6:** The concentration and coordination index for all the experts.

Item	Mean	Percentage of full score (%)	Sum of rank	Coefficient of variation
Chest fullness/chest pain	4.43	55.9	146	0.161
Excessive phlegm	4.16	41.9	171	0.198
Abdominal fullness	4.20	40.9	167	0.181
Obesity	4.12	31.2	175	0.182
Anorexia	3.82	22.6	203	0.222
Nausea and vomiting	3.63	17.2	220	0.236
head heaviness	4.11	29.0	176	0.177
Vertigo	3.95	29.0	191	0.237
Somnolence	3.72	18.3	212	0.232
Physical heaviness	4.31	45.2	157	0.171
Fatigue	3.94	32.3	172	0.230
Pale tongue	3.39	16.1	243	0.298
Greasy fur	4.76	77.4	127	0.095
Slippery pulse	4.54	61.3	136	0.140
Soft pulse	3.48	12.9	234	0.267
Stringy Pulse	3.59	20.4	224	0.270

**Table 7:** The items of phlegm turbidity syndrome for CHD.

Symptoms	Definition	None	Mild	Moderate	Severe
Chest fullness/Chest pain	Have suppression or choking sensation in the chest. Have pain in the center or partial side of the chest.	0	1	2	3
Excessive phlegm	Abundance of phlegm.	0	1	2	3
Physical heaviness	Physically heavy and have mobility problems.	0	1	2	3
Fullness	Feel full and stuffy in the stomach.	0	1	2	3
Anorexia	Loss of appetite.	0	1	2	3
Obesity	Abnormally fat.	0	1	2	3
Head heaviness	The head is pounded, feeling like being wrapped.	0	1	2	3
Vertigo	Feeling dizzy, vision blurred.	0	1	2	3
Greasy fur	Thin or thick greasy coatings cover the tongue.	0	1	2	3
Fatigue	Feeling tired, and lack of energy.	0	1	2	3
Slippery pulse	The pulse is fluent and smooth, feeling like beads rolling in a plate.	0	1	2	3
Stringy pulse	The pulse is straight and long, feeling like a string.	0	1	2	3

## Data Availability

Rong Xie and Xiantao Li had full access to all of the data in the study and take responsibility for the integrity of the data and the accuracy of the data analysis.
